# *MDR1* causes resistance to the antitumour drug miltefosine

**DOI:** 10.1054/bjoc.2001.1776

**Published:** 2001-05

**Authors:** M Rybczynska, R Liu, P Lu, F J Sharom, E Steinfels, A Di Pietro, M Spitaler, H Grunicke, J Hofmann

**Affiliations:** Institute of Medical Chemistry and Biochemistry, University of Innsbruck, Fritz-Pregl-Str. 3, A-6020 Innsbruck, Austria; Department of Chemistry and Biochemistry, University of Guelph, 50 Stone Road East, Guelph, ON, N1G 2W1, Canada; Institute of Protein Biology and Chemistry, UMR-CNRS 5086, Passage du Vercors 7, F-69367 Lyon, France

**Keywords:** miltefosine, hexadecylphosphocholine, multidrug resistance;*MDR1*

## Abstract

Miltefosine (hexadecylphosphocholine) is used for topical treatment of breast cancers. It has been shown previously that a high percentage of breast carcinomas express *MDR1* or *MRP*. We investigated the sensitivity of *MDR1* -expressing cells to treatment with miltefosine. We show that cells overexpressing *MDR1* (NCI/ADR-RES, KB-8-5, KB-C1, CCRF/VCR1000, CCRF/ADR5000) were less sensitive to miltefosine treatment when compared to the sensitive parental cell lines. HeLa cells transfected with *MDR1* exhibited resistance to the compound, indicating that expression of this gene is sufficient to reduce the sensitivity to miltefosine. The resistance of *MDR1* -expressing cells to miltefosine was less pronounced than that to adriamycin or vinblastine. Expression of *MDR2* did not correlate with the resistance to miltefosine. As shown by a fluorescence quenching assay using MIANS-labelled P-glycoprotein (PGP), miltefosine bound to PGP with a K _d_ of approximately 7 μM and inhibited PGP-ATPase activity with an IC _50_ of approximately 35 μM. Verapamil was not able to reverse the resistance to miltefosine. Concentrations of miltefosine up to approximately 60 μM stimulated, whereas higher concentrations inhibited the transport of [^3^H]-colchicine with an IC _50_ of approximately 297 μM. Binding studies indicated that miltefosine seems to interact with the transmembrane domain and not the cytosolic nucleotide-binding domain of PGP. These data indicate that expression of *MDR1* may reduce the response to miltefosine in patients and that this compound interacts with PGP in a manner different from a number of other substrates. © 2001 Cancer Research Campaign www.bjcancer.com


*British Journal of Cancer* (2001) **84**(10), 1405–1411
© 2001 Cancer Research Campaign
doi: 10.1054/ bjoc.2001.1776, available online at http://www.idealibrary.com on IDEAL®

### *MDR1* causes resistance to the antitumour drug miltefosine

**M Rybczynska[1], R Liu[2], P Lu[2], FJ Sharom[2], E Steinfels[3], A Di Pietro[3], M Spitaler[1], H Grunicke[1] and J Hofmann[1]**

[1]Institute of Medical Chemistry and Biochemistry, University of Innsbruck, Fritz-Pregl-Str. 3, A-6020 Innsbruck, Austria; [2]Department of Chemistry and Biochemistry, University of Guelph, 50 Stone Road East, Guelph, ON, N1G 2W1, Canada; [3]Institute of Protein Biology and Chemistry, UMR-CNRS 5086, Passage du Vercors 7, F-69367 Lyon, France

**Summary** Miltefosine (hexadecylphosphocholine) is used for topical treatment of breast cancers. It has been shown previously that a high percentage of breast carcinomas express *MDR1* or *MRP*. We investigated the sensitivity of *MDR1*-expressing cells to treatment with miltefosine. We show that cells overexpressing *MDR1* (NCI/ADR-RES, KB-8-5, KB-C1, CCRF/VCR1000, CCRF/ADR5000) were less sensitive to miltefosine treatment when compared to the sensitive parental cell lines. HeLa cells transfected with *MDR1* exhibited resistance to the compound, indicating that expression of this gene is sufficient to reduce the sensitivity to miltefosine. The resistance of *MDR1*-expressing cells to miltefosine was less pronounced than that to adriamycin or vinblastine. Expression of *MDR2* did not correlate with the resistance to miltefosine. As shown by a fluorescence quenching assay using MIANS-labelled P-glycoprotein (PGP), miltefosine bound to PGP with a $K_d$ of approximately 7 μM and inhibited PGP-ATPase activity with an $IC_{50}$ of approximately 35 μM. Verapamil was not able to reverse the resistance to miltefosine. Concentrations of miltefosine up to approximately 60 μM stimulated, whereas higher concentrations inhibited the transport of [$^3$H]-colchicine with an $IC_{50}$ of approximately 297 μM. Binding studies indicated that miltefosine seems to interact with the transmembrane domain and not the cytosolic nucleotide-binding domain of PGP. These data indicate that expression of *MDR1* may reduce the response to miltefosine in patients and that this compound interacts with PGP in a manner different from a number of other substrates. © 2001 Cancer Research Campaign http://www.bjcancer.com

**Keywords**: miltefosine; hexadecylphosphocholine; multidrug resistance; *MDR1*

Phospholipid analogues are a new class of drugs, which exhibit broad antineoplastic activity (Berdel, 1991; Brachwitz and Vollgraf, 1995). Miltefosine represents the first of these compounds used in the clinic (Berdel, 1991; Brachwitz and Vollgraf, 1995). It is approved in several countries for the topical treatment of skin metastases resulting from breast cancers (Hilgard et al, 1993). The exact mechanism of action responsible for the antitumour activity of miltefosine is not yet known (Berdel, 1991; Hilgard et al, 1993; Brachwitz and Vollgraf, 1995).

A major problem in the treatment of tumours with antitumour agents is the existence of tumour cell populations with intrinsic or acquired resistance (Goldie and Coldman, 1984). For the clinical use of miltefosine, the following questions are important: (i) why are tumour cells refractory to the compound, and (ii) are tumours, that are resistant to antitumor agents used in the treatment of breast cancer also cross-resistant to miltefosine?

Resistance to a spectrum of antitumour drugs is frequently associated with the expression of *MDR1*, *MRP1*, *BCRP* or *LRP* genes belonging to the ATP-binding cassette superfamily of membrane transport proteins (Gottesman and Pastan, 1993; Scheffer et al, 1995; Lautier et al, 1996; Doyle et al, 1998). Approximately 40 to 50% of primary breast carcinomas express *MDR1* (Trock et al, 1997). Recently, we have shown that *MDR1*-expressing cells are cross-resistant to the phospholipid analogue ilmofosine (Hofmann et al, 1997). In view of the relevance of miltefosine for treatment of cancers, we investigated the association of *MDR1*-mediated resistance and the low sensitivity of tumour cells to miltefosine.

*Received 20 March 2000*
*Revised 13 February 2001*
*Accepted 15 February 2001*

*Correspondence to:* J Hofmann

### MATERIALS AND METHODS

### Drugs

Miltefosine was from ASTA Medica (Frankfurt, Germany). A 10 mM stock solution in 20 mM Tris-HCl (pH 7.4) was used for further dilutions. Vinblastine and adriamycin were from Sigma, Munich, Germany. The MTT-assay kit was obtained from Boehringer-Mannheim, Mannheim, Germany. [$^3$H]-colchicine (15–25 Ci mmol) was purchased from DuPont NEN (Boston, MA, USA), and MIANS was obtained from Molecular Probes (Eugene, OR, USA).

### Tissue culture

CCRF-CEM (human lymphoblastoid cells), the multidrug resistant sublines CCRF/VCR1000, CCRF/ADR5000 (Kimmig et al, 1990), HeLa (human epitheloid cervix carcinoma) and 2 multidrug resistant sublines (HeLa-MDR1-G185, HeLa-MDR1-V185) were grown in RPMI 1640 medium. MCF7 (human breast adenocarcinoma) cells, the multidrug resistant line NCI/ADR-RES, KB-3-1 cells (human oral epidermoid carcinoma), and the multidrug resistant sublines KB-8-5 and KB-C1 (Akiyama et al, 1985), were grown in Dulbecco's modified Eagle's medium (4.5 g glucose$^{-1}$). The NCI/ADR-RES cell line was distributed by the NCI and was believed to be a MCF7-derived resistant subline. However, recently it has been revealed that it is not derived from MCF7.

Multidrug resistant CH$^R$B30 Chinese hamster ovary cells (Ling and Thompson, 1974) were grown in α-minimal essential medium. The medium was supplemented with 10% fetal calf serum, 2 mM glutamine and 50 μg ml$^{-1}$ gentamycin. Multidrug-resistant stock cultures were grown in presence of the following drugs (except at the time of experiments): NCI/ADR-RES: 10 μg adriamycin ml$^{-1}$; CCRF/VCR1000: 1 μg vincristine sulphate ml$^{-1}$; CCRF/ADR5000: 5 μg adriamycin ml$^{-1}$; HeLa-MDR1-G185: 100 nM vinblastine; HeLa-MDR1-V185: 240 ng colchicine ml$^{-1}$; KB-8-5: 10 ng colchicine ml$^{-1}$; KB-C1: 1 μg colchicine ml$^{-1}$; CH$^R$B30:30 μg colchicine ml$^{-1}$.

The two multidrug-resistant *MDR1*-overexpressing HeLa cell lines were obtained by transfection of human HeLa S3 (HeLa-WT) cervix carcinoma cells with a MDR1 wild-type gene construct (HeLa-MDR1-G185) and with a mutation in codon 185 (Gly-Val, kindly provided by Dr M M Gottesman, HeLa-MDR1-V185), respectively (Kane et al, 1989). Following transfection, HeLa-MDR1-G185 cells were grown in the presence of vinblastine (100 nM) and HeLa-MDR1-V185 in the presence of colchicine (240 ng ml$^{-1}$). One clone of each cell line was taken for further cultivation. MDR1-mRNA expression was controlled by reverse transcriptase PCR (Hofmann et al, 1997). Wild-type and mutant genes were controlled by sequencing (Spitaler et al, 1998).

Dose–response curves for calculations of the $IC_{50}$ values (Table 1) were obtained by plating the cells in 96-well plates. Following an incubation period of 4 hours, the drugs were added and the cells were exposed to the drugs continuously for 72 hours. Subsequently, cell proliferation was detected by the MTT assay (Mosman, 1983). The $IC_{50}$ values were calculated using CalcuSyn software from Biosoft, Cambridge, UK.

### Detection of MDR1 and MDR2 mRNA levels

For detection of the mRNA levels, total RNA was isolated using RNAzol (Biotexs Laboratories Inc, Houston, TX, USA). Synthesis of cDNA and amplification of the *MDR1*-mRNA by polymerase chain reaction was performed as described (Noonan et al, 1990). Primers for the amplification of the MDR2-mRNA were: 2061–2083 (5′-TGT CAG AAG AGC CTT GAT GTG G-3′) and 2193–2215 (5′-TGG CAA TGG CAC ATA CTG TTC C-3′). β-Microglobulin was used to control the correct amount of RNA in the experiments (Noonan et al, 1990). Amplifications (30 cycles) were performed with a denaturation temperature of 94˚C (35 seconds), an annealing temperature of 57˚C (30 seconds), and an extension temperature of 73˚C (1 minute). Starting with cycle 16, the time for synthesis was extended (5 seconds per cycle). The reaction products were separated on a 10% polyacrylamide gel and stained with ethidium bromide.

### MIANS-PGP quenching assay

Binding of miltefosine to P-glycoprotein (PGP) was carried out using fluorescence quenching, as described previously for drugs, chemosensitizers and hydrophobic peptides (Liu and Sharom, 1996; Sharom et al, 1998a, 1998b). Highly purified PGP, labelled with MIANS (Liu and Sharom, 1996) was titrated with miltefosine and quenching of the fluorescence emission at 420 nm was monitored. The dissociation constant $K_d$ for binding was estimated by fitting the data to an equation describing interaction with a single class of binding site.

### PGP ATPase activity

The ATPase activity of P-glycoprotein in CH$^R$B30 plasma membrane was measured as described previously (Doige et al, 1992) by detection of the release of inorganic phosphate from ATP, using a colorimetric method. Membrane vesicles (1–2 μg of protein) in buffer containing 2 mM ATP and 5 mM $Mg^{2+}$ were pre-incubated with miltefosine for 5 minutes before initiation of the assay by addition of ATP.

### PGP-mediated [$^3$H]-colchicine transport

ATP-dependent uptake of [$^3$H]-colchicine into CH$^R$B30 plasma membrane vesicles was determined by rapid filtration as outlined earlier (Sharom et al, 1996), in the presence of increasing concentrations of miltefosine. Colchicine uptake was calculated as percent relative to a control in the absence of drug.

### RESULTS

### Effects of miltefosine on *MDR1*-expressing cells

It has been demonstrated that the resistant sublines shown in Table 1 overexpress MDR1 (Figure 1; Akiyama et al, 1985; Kimmig et al, 1990; Hofmann et al, 1997). The resistance of the cell lines employed in this study to vinblastine and adriamycin (as attested by $IC_{50}$ values and factors of resistance) is shown in Table 1. All MDR1-expressing sublines exhibited cross-resistance to miltefosine. The resistance to the compound was less pronounced than that to vinblastine or adriamycin (Table 1). Resistance to miltefosine was also observed in a cell line transfected with wild-type or mutant PGP. This supports the notion that expression of *MDR1* is sufficient to elicit resistance to miltefosine, and that it is not due to additional resistance mechanisms possibly induced during selection with adriamycin, vincristine or colchicine. Compared to the degree of resistance to vinblastine or adriamycin, the KB-C1 cell line exhibits low resistance to miltefosine (Figure 1). PGP expressed in KB-C1 cells harbours a mutation in position 185 (glycine to valine) (Choi et al, 1988; Safa et al, 1990). Compared to wild-type PGP, the mutated PGP exhibited different substrate specificity for drugs (Choi et al, 1988; Safa et al, 1990) and differences in the sensitivity to reversing agents (Cardarelli et al, 1995). We investigated whether this mutation might influence the resistance to miltefosine. In a HeLa subline transfected with mutant PGP (HeLa-MDR1-V185) the profile of resistance to vinblastine and adriamycin was altered. Despite slightly higher expression of MDR1 in the HeLa-MDR1-V185 compared to the HeLa-MDR1-G185 cell line (Figure 1), the resistance to vinblastine was decreased (Table 1). This is in accordance with results published previously (Choi et al, 1988; Safa et al, 1990). However, this mutation did not alter the resistance to miltefosine significantly (Table 1; HeLa-MDR1-G185 = 8.3-fold, HeLa-MDR1-V185 = 9.6-fold).

### MDR2-expression

It has been reported that the MDR2-encoded PGP transports phospholipids out of the cell (Smit et al, 1993; Ruetz and Gros, 1994). Thorgeirsson et al (1991) proposed a possible mechanism for co-induction of the *MDR1* and *MDR2* genes. If both genes are co-expressed, *MDR2* might be responsible for the resistance to

 *© 2001 Cancer Research Campaign*

**Table 1** $IC_{50}$ values of vinblastine, adriamycin and miltefosine. $IC_{50}$ was obtained from dose–response curves to the drugs as described in 'Materials and Methods'. The means of at least three independent experiments, in which duplicate determinations were taken within each experiment, are indicated. The resistance factor in brackets, indicating the resistance compared with the parental cell line, is calculated by the $IC_{50}$-resistant/$IC_{50}$-sensitive ratio. MDR1-expressing cell lines and the resistance factors are printed in bold

| Cell line | Vinblastine (nM) | | Adriamycin (nM) | | Miltefosine (μM) | |
|---|---|---|---|---|---|---|
| MCF7 | 5.5 (± 0.6) | | 155.4 (± 18.6)) | | 34.6 (± 11.7) | |
| **NCI/ADR-RES** | 116.9 (± 10.7) | ***(21.2)*** | 1352.8 (± 121.7) | ***(8.70)*** | 69.8 (± 4.5) | ***(2.0)*** |
| KB-3-1 | 1.8 (± 0.2) | | 46.6 (± 9.3) | | 2.5 (± 0.3) | |
| **KB-8-5** | 28.4 (± 5.2) | ***(15.9)*** | 390.7 (± 57.4) | ***(8.3)*** | 3.2 (± 0.3) | ***(1.3)*** |
| **KB-C1** | 66.5 (± 22.5) | ***(37.3)*** | 2569.5 (± 128.4) | ***(55.1)*** | 4.5 (± 0.3) | ***(1.8)*** |
| HeLa-WT | 7.3 (± 3.7) | | 231.0 (± 9.0) | | 6.8 (± 0.9) | |
| **HeLa-MDR1-G185** | 251.0 (± 15.1) | ***(34.3)*** | 25650.0 (± 3190.0) | ***(111.0)*** | 57.0 (± 18.2) | ***(8.3)*** |
| **HeLa-MDR1-V185** | 91.9 (± 11.3) | ***(12.5)*** | 16080.0 (± 5949.6) | ***(69.6)*** | 65.8 (± 4.4) | ***(9.6)*** |
| CCRF/CEM | 2.0 (± 0.1) | | 21.6 (± 1.5) | | 4.9 (± 1.2) | |
| **CCRF/VCR1000** | 492.0 (± 61.5) | ***(246.0)*** | 1459.0 (± 91.1) | ***(67.5)*** | 29.2 (± 2.7) | ***(5.9)*** |
| **CCRF/ADR5000** | 2420 (± 387.2) | ***(1210.0)*** | 846.0 (± 50.7) | ***(39.1)*** | 45.3 (± 12.7) | ***(7.4)*** |

miltefosine. In order to investigate whether *MDR2* might be involved in the resistance to miltefosine, we detected the expression of the *MDR2* gene. As shown in Figure 1, there was no association between the resistance to miltefosine and the expression of the *MDR2* gene. For example, the sensitive MCF7 and the resistant NCI/AD-RRES cell lines express *MDR2* to a similar extent. On the other hand, neither sensitive HeLa-WT, nor the multidrug-resistant *MDR1*-transfected HeLa cell lines express *MDR2*. Drug-sensitive CCRF/CEM cells express low levels of *MDR2*, whereas the miltefosine-resistant CCRF/ADR5000 show no *MDR2*-expression (Figure 1). Thus, *MDR2*-expression does not correspond to the resistance to miltefosine. In contrast, there is a clear association between the expression of *MDR1* and the resistance to miltefosine (Table 1, Figure 1).

### Interaction of miltefosine with PGP

In order to investigate whether miltefosine binds to PGP, we carried out a fluorescence quenching assay using highly purified protein isolated from $CH^RB30$ cells. Binding of a wide variety of substrates to PGP, including drugs, chemosensitizers, and hydrophobic peptides, leads to saturable fluorescence quenching of highly purified PGP labelled with the fluorophore MIANS at two conserved cysteine residues within the Walker A motifs of the nucleotide-binding domains (Liu and Sharom, 1996; Sharom et al, 1998a, 1998b). The quenching curve can be used to estimate the dissociation constant, $K_d$, which is a measure of the affinity of binding (Liu and Sharom, 1996; Sharom et al, 1998a, 1998b). To date, $K_d$ values have been measured covering the range 250 μM to 25 nM (Sharom et al, 1998a, 1999). As shown in Figure 2, miltefosine bound to purified MIANS-PGP with a $K_d$ of approximately 7 μM, indicating that the compound is a relatively high-affinity substrate for PGP. Drugs and chemosensitizers that interact with PGP can either stimulate or inhibit the basal ATPase activity of PGP (Thorgeirsson et al, 1991; Doige et al, 1992; Gottesman and Pastan, 1993; Sharom et al, 1995; Sharom, 1997). In plasma membrane vesicles from the highly drug-resistant CHO cell line $CH^RB30$, miltefosine inhibited the ATPase activity with an $IC_{50}$ of 35 μM (Figure 3). In addition to the data shown in Figure 2, this is a further indication of an interaction between miltefosine and PGP. Miltefosine was also found to bind to the *Bacillus subtilis* ABC transporter YvcC with a $K_d$ of 2–3 μM (data not shown).

In order to investigate whether miltefosine interacts directly with the cytosolic NBD of PGP, murine NBD2 was purified and

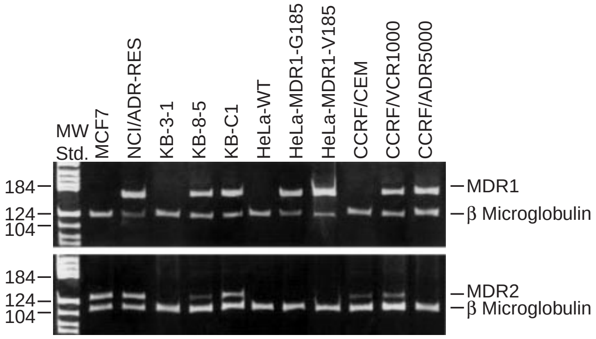

**Figure 1** Detection of MDR1 and MDR2 mRNA levels. Isolation of RNA, cDNA synthesis and amplification by PCR was performed as described in Materials and Methods. β-Microglobulin was employed as a control for the correct amount of RNA used in the experiment

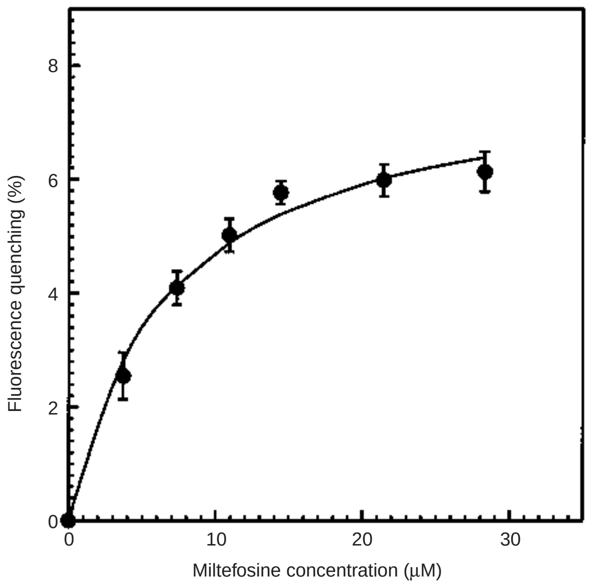

**Figure 2** Quenching of MIANS-PGP by miltefosine. Increasing concentrations of miltefosine were added to highly purified PGP labelled with MIANS. The percent quenching of the fluorescence emission at 420 nm (deltaF/F0) was calculated relative to MIANS-labelled PGP in the absence of miltefosine. The quenching data (means ±range, *n* = 2) were fitted to an equation describing interaction with a single binding site

*© 2001 Cancer Research Campaign*

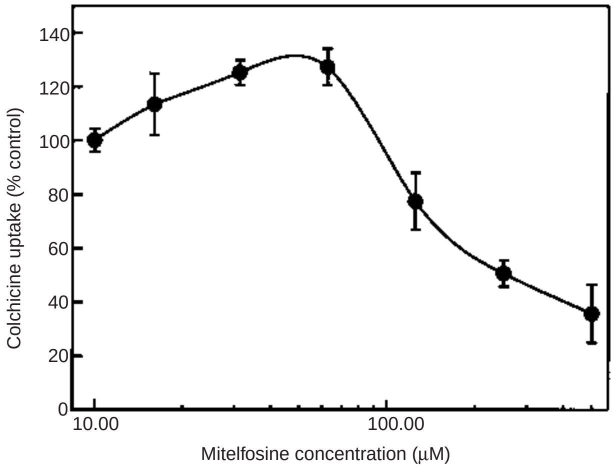

**Figure 3** Modulation of PGP-ATPase activity by miltefosine. ATPase activity was measured in plasma membranes from MDR1-expressing $CH^RB30$ cells as described in Experimental Procedures. Data are presented as the percentage of control ATPase activity in the absence of miltefosine (means ± SEM, $n = 3$)

fluorescence quenching was determined (Conseil et al, 1998). A wide range of miltefosine concentrations (2 μM–2 mM) did not show any significant quenching. The binding site of the antiprogestin RU486 is close to the ATP-binding site (Conseil et al, 1998). The $K_d$ for RU486 in the absence of miltefosine, 17.9 ± 2.6 μM, was not markedly changed upon addition of miltefosine up to 320 μM (not shown here). This is an indication that the cytosolic NBDs do not contain the main binding site for miltefosine. The interaction site is therefore likely to be located within the transmembrane domain. The main binding site for steroids and other PGP transport substrates has also been found to be located in the transmembrane regions (Sharom, 1997; Vo and Gruol, 1999).

### Combination of verapamil and miltefosine

If the resistance of *MDR1*-expressing cells to miltefosine is due to PGP-mediated efflux, verapamil should block the efflux and thereby reverse the resistance to miltefosine. Usually 5 μM verapamil is sufficient to reverse the resistance to antitumor drugs. However, in multidrug-resistant HeLa cells (HeLa-MDR1-G185), 10 μM verapamil did not reverse resistance (Figure 4). Verapamil also did not enhance the antiproliferative activity of miltefosine in drug-sensitive HeLa-WT cells. In HeLa-MDR1-V185 harbouring a mutant PGP, the combination verapamil/miltefosine is slightly more effective than in drug-sensitive HeLa-WT and HeLa-MDR1-G185. In HeLa-MDR1-V185 this effect is not synergistic, but is at best additive. For comparison, the synergistic effect of vinblastine in combination with verapamil in HeLa-MDR1-G185 cells is shown (Figure 5). Thus, although miltefosine seems to be transported by PGP, the efflux cannot be blocked by verapamil (Figure 4). This is an indication that miltefosine seems to show anomalous behaviour compared to other substrates.

### Modulation of the colchicine transport by miltefosine

If miltefosine interacts directly with PGP, it should modulate the transport of other drugs by the protein. Many PGP substrates and chemosensitizers inhibit colchicine transport into plasma membrane vesicles from MDR cells, or into reconstituted proteoliposomes containing PGP (Doige and Sharom, 1992; Sharom et al, 1993, 1995; Sharom, 1997). A good correlation has been found between the $K_d$ value for many drugs, chemosensitizers and peptides (as determined by MIANS-PGP quenching), and the $IC_{50}$ for inhibition of PGP-mediated [$^3$H]colchicine uptake into $CH^RB30$ plasma membrane vesicles (Sharom et al, 1998a, 1998b). Some peptides and other compounds have been found to stimulate the transport of drugs, likely via positive allosteric effects (Sharom et al, 1996; Shapiro and Ling, 1997; Shapiro et al, 1999). Miltefosine at concentrations up to 60 μM led to a stimulation of colchicine transport into $CH^RB30$ plasma membrane vesicles (Figure 6). The observed activation in the range of 27% was highly reproducible in each experiment. This activation of colchicine transport can be explained by a positive allosteric interaction between two distinct, but possibly overlapping, substrate-binding sites for miltefosine and colchicine. Much higher concentrations of miltefosine led to inhibition of colchicine transport, with an $IC_{50}$ of approximately 300 μM. The inhibition of transport at high concentrations of miltefosine is probably due to non-specific detergent-like effects, as were observed previously for certain membrane-active peptides (Sharom et al, 1995).

### DISCUSSION

We have investigated the susceptibility of human tumour cell lines to treatment with the phospholipid analogue miltefosine. We found that the expression of *MDR1* elicits resistance to this compound, although it was less pronounced than that to adriamycin or vinblastine. In view of the fact that breast cancers frequently

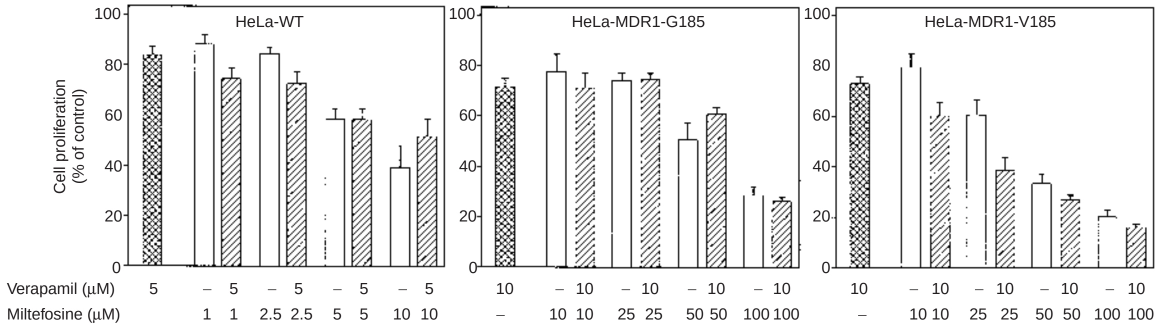

**Figure 4** Combination of verapamil and miltefosine. HeLa cells were grown in presence of verapamil, miltefosine or a combination of both for 72 hours. Cell proliferation was determined by the MTT assay. The means (± SEM) of 3 independent experiments, in which duplicate determinations were taken within each experiment, are indicated

© 2001 Cancer Research Campaign

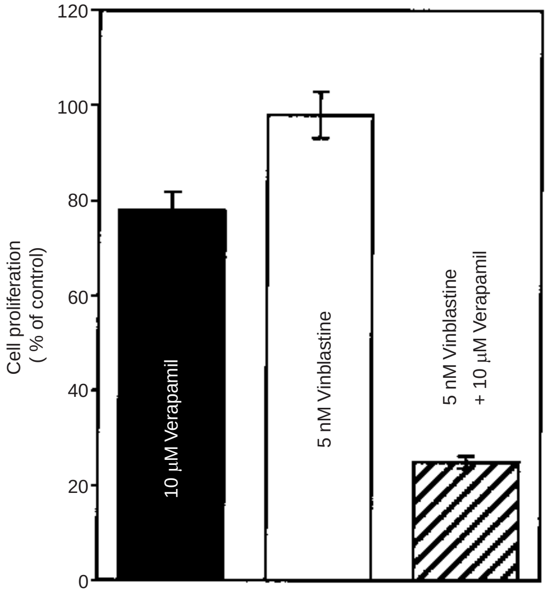

**Figure 5** Combination of verapamil and vinblastine. HeLa-MDR1-G185 cells were grown in the presence of verapamil, vinblastine or a combination of both for 72 hours. The means of two independent experiments (± SD), in which 4 determinations were taken within each experiment, are indicated

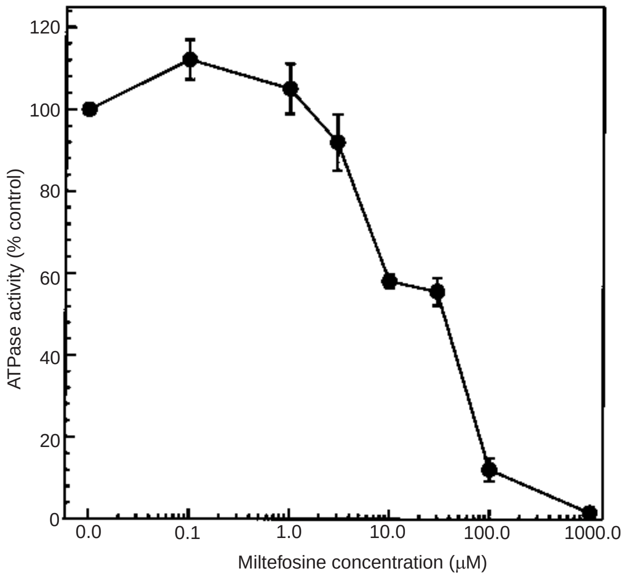

**Figure 6** Modulation of colchicine transport by miltefosine. Equilibrium uptake of [$^3$H]-colchicine into plasma membrane vesicles of MDR1-expressing CH$^R$B30 cells was measured at 22˚C in the presence of 1 mM ATP and an ATP-regenerating system. Data represent the percent of control (in absence of miltefosine). The means of 3 different experiments (± SEM) are indicated express *MDR1* (Trock et al, 1997), the observed resistance may suffice to reduce the efficacy of miltefosine, or to cause treatment failures. Breast cancers are, among others, treated with drugs transported by PGP, such as adriamycin, vinblastine, vincristine, etoposide or taxol. Our results show that tumours resistant to these compounds, due to the expression of *MDR1*, also exhibit reduced sensitivity to miltefosine. Cross-resistance of multidrug-resistant cells to miltefosine was observed previously, when multidrug resistance was induced with adriamycin, but not with colchicine (Himmelmann et al, 1990). Our results are in agreement with these data. The resistance to miltefosine of KB-8-5 and KB-C1 cells in which the resistance was induced by colchicine is very modest (Table 1). Resistance to miltefosine is more pronounced in adriamycin-induced cells (NCI/ADR-RES, CCRF/ADR5000) compared to the level of resistance to adriamycin. It is conceivable that cross-resistance to miltefosine may develop not only via induction of *MDR1* expression by adriamycin, but also by other mechanisms. However, we show here that two HeLa cell lines in which the resistance was obtained by transfection with *MDR1* also exhibited resistance to miltefosine (Table 1). These results demonstrate that expression of *MDR1* is sufficient to elicit resistance to the compound. A mutation at position 185 that alters the substrate specificity for several compounds does not influence the miltefosine resistance significantly.

One possible explanation for the cross-resistance of multidrug-resistant cells to miltefosine would be that the compound is transported by PGP. This assumption is confirmed here by the fact that the expression of *MDR1* correlates with the resistance to miltefosine. mRNA levels of *MDR2* are not related to the miltefosine resistance. One approach to investigate whether a particular compound is a substrate of PGP is the determination of the quenching of the PGP-bound fluorescence probe MIANS (Liu and Sharom, 1996; Sharom et al, 1998a, 1998b). Using this assay, interaction of miltefosine was observed with PGP with a $K_d$ value of approximately 7 μM (Figure 2). Interaction with comparable affinity was observed with the bacterial ABC transporter YvcC. As indicated by experiments with NBD2 of murine PGP, miltefosine does not interact with the cytosolic ATP-binding site. A likely binding site may therefore be a drug/modulator-binding site within the transmembrane domain. Miltefosine also inhibited the ATPase activity of PGP (Figure 3). These data illustrate that miltefosine does interact with PGP and the resistance to miltefosine seems to be due to efflux of the compound. Abulrob and Gumbletan (1999) reported that in *MDR2*-negative KB and MCF cells in which the expression of *MDR1* was induced, the intracellular accumulation of a fluorescently-labelled phosphatidylcholine analogue was reduced as compared to the sensitive cells. These data confirm that the *MDR1*-encoded PGP is able to transport phospholipids. This proposal is supported by the recent report from one of our laboratories that PGP reconstituted into proteoliposomes acts as a flippase for a number of fluorescent phospholipids (Romsicki and Sharom, 2001).

As shown in Figure 4, the multidrug resistance modulator verapamil was not able to reverse the resistance to miltefosine. Recently, it has been shown that cells selected for resistance by the addition of miltefosine also express *MDR1*, indicating a connection between PGP and resistance to miltefosine. This resistance also could not be reversed by verapamil (Fu et al, 1999), which indicates that miltefosine does not interact with the verapamil-binding site. In agreement with this hypothesis is the observation that miltefosine increased the transport of [$^3$H]-colchicine (Figure 6). Most PGP substrates show a good correlation between the $K_d$ value and the $IC_{50}$ for inhibition of the ATP-dependent [$^3$H]-colchicine uptake. Miltefosine exhibits unusual behaviour in that it activates transport at concentrations up to 60 μM. Although higher concentrations inhibit the colchicine uptake, such high concentrations of miltefosine may lead to detergent-like effects as observed previously for membrane-active peptides (Sharom et al, 1993). An explanation for these unusual results might be that miltefosine does not interact, as do many other substrates, with a site of PGP

that leads to transport inhibition, but rather with a site that leads to allosteric activation of drug transport. To date, over 60 PGP substrates and modulators have been found to inhibit colchicine transport into membrane vesicles (Sharom et al, 1999). A few compounds have been found to stimulate colchicine transport (Lu and Sharom, unpublished data). Results similar to those with miltefosine were obtained with the synthetic peptide NAc-LLY-amide which is a substrate of PGP, but activates colchicine transport (Sharom et al, 1998b). NAc-LLY-amide also did not compete with azidopine photolabelling. More recently, Shapiro and co-workers have shown that transport of the fluorescent PGP substrates rhodamine 123 and Hoechst 3342 in $CH^RB30$ plasma membrane vesicles was stimulated by several other compounds in a positive allosteric fashion (Shapiro and Ling, 1997; Shapiro et al, 1999).

*MDR1*-expressing cells have also been shown to be resistant to the phospholipid analogue ilmofosine. This compound slightly increased the photolabelling of PGP by azidopine (Hofmann et al, 1997). This illustrates that ilmofosine, like miltefosine, increases the interactions of PGP with certain substrates. From the facts that i) ilmofosine enhanced azidopine photolabelling (Hofmann et al, 1997), ii) the PGP-modulator dexniguldipine-HC1 did not reverse the resistance to ilmofosine (Hofmann et al, 1997), iii) verapamil did not reverse the resistance to miltefosine (Fu et al, 1999), and iv) cells resistant to miltefosine, in addition to *MDR1*, also expressed elevated levels of bcl-2, it was concluded that the phospholipid analogues ilmofosine (Hofmann et al, 1997) and miltefosine (Fu et al, 1999) are not substrates of PGP. However, these conclusions were not based on direct experiments designed to answer this question. The results obtained by the experiments shown contradict these conclusions, and show direct interaction of miltefosine with PGP.

### ACKNOWLEDGEMENTS

This work was supported by grant P10664-MED to J Hofmann from the Austrian Science Fund and by a grant from the National Cancer Institute of Canada, with funds provided by the Canadian Cancer Society to F J Sharom. E Steinfels was recipient of a fellowship from the Ligue Nationale contre le Cancer (comité de Haute-Savoie).

The current address of M Rybczynska is: Karol Marcinkowskis University of Medical Sciences, Biochemistry and Medical Analysis, Przybyszewskiego 49, Poznan 60 335, Poland.

### REFERENCES

Abulrob AG and Gumbleton M (1999) Transport of phosphatidylcholine in MDR3-negative epithelial cell lines via drug-induced MDR1 P-glycoprotein. *Biochem Biophys Res Commun* **262**: 121–126

Akiyama S, Fojo A, Hanover JA, Pastan I and Gottesman MM (1985) Isolation and genetic characterization of human KB cell lines resistant to multiple drugs. *Somatic Cell Mol Genet* **11**: 117–126

Berdel WE (1991) Membrane-interactive lipids as experimental anticancer drugs. *Br J Cancer* **64**: 208–211

Brachwitz H and Vollgraf C (1995) Analogs of alkyllysophospholipids: chemistry, effects on the molecular level and their consequences for normal and malignant cells. *Pharmacol Ther* **66**: 39–82

Cardarelli CO, Aksentijevich I, Pastan I and Gottesman MM (1995) Differential effects of P-glycoprotein inhibitors on NIH3T3 cells transfected with wild-type (G185) or mutant (V185) multidrug transporters. *Cancer Res* **55**: 1086–1091

Choi K, Chen C, Kriegler M and Roninson IB (1988) An altered pattern of cross-resistance in multidrug resistant human cells results from spontaneous mutations in the mdr1 (P-glycoprotein) gene. *Cell* **53**: 519–529

Conseil G, Baubichon-Cortay H, Dayan G, Jault J-M, Barron D and Di Pietro A (1998) Flavonoids: a class of modulators with bifunctional interactions at vicinal ATP-and steroid-binding sites on mouse P-glycoprotein. *Proc Natl Acad Sci USA* **95**: 9831–9836

Doige CA and Sharom FJ (1992) Transport properties of P-glycoprotein in plasma membrane vesicles from multidrug-resistant Chinese hamster ovary cells. *Biochim Biophys Acta* **1109**: 161–171

Doige CA, Yu X and Sharom FJ (1992) ATPase activity of partially purified P-glycoprotein from multidrug-resistant Chinese hamster ovary cells. *Biochim Biophys Acta* **1109**: 149–160

Doyle AL, Yang W, Abruzzo LV, Krogmann T, Gao Y, Rishi AK and Ross DG (1998) A multidrug resistance transporter from MCF-7 breast cancer cells. *Proc Natl Acad Sci USA* **95**: 15665–15670

Fu D, Shi Z and Wang Y (1999) Bcl-2 plays a key role instead of mdr1 in the resistance to hexadecylphosphocholine in human epidermoid tumor cell line KB. *Cancer Lett* **142**: 147–153

Goldie JH and Coldman AJ (1984) The genetic origin of drug resistance in neoplasms: implications for systemic therapy. *Cancer Res* **44**: 3643–3653

Gottesman MM and Pastan I (1993) Biochemistry of multidrug resistance mediated by the multidrug transporter. *Annu Rev Biochem* **62**: 385–427

Hilgard P, Klenner T, Stekar J and Unger C (1993) Alkylphosphocholines: a new class of membrane-active anticancer agents. *Cancer Chemother Pharmacol* **32**: 90–95

Himmelmann AW, Danhauser-Riedl S, Steinhauser G, Busch R, Modest EJ, Noseda A, Rastetter J, Vogler WR and Berdel WE (1990) Cross-resistance pattern of cell lines selected for resistance towards different cytotoxic drugs to membrane-toxic phospholipids in vitro. *Cancer Chemother Pharmacol* **26**: 437–443

Hofmann J, Utz I, Spitaler M, Hofer S, Rybczynska M, Beck WT, Herrmann DBJ and Grunicke H (1997) Resistance to the new anti-cancer phospholipid ilmofosine (BM 41 440). *Br J Cancer* **76**: 862–869

Kane SE, Reinhard DH, Fordis MC, Pastan I and Gottesman MM (1989) A new vector using the human multidrug resistance gene as a selectable marker enables overexpression of foreign genes in eukaryotic cells. *Gene* **84**: 439–446

Kim CH, Gollapudi S, Lee T and Gupta S (1997) Altered expression of the genes regulating apoptosis in multidrug resistant human myeloid leukemia cell lines overexpressing MDR1 or MRP gene. *Int J Oncol* **11**: 945–950

Kimmig A, Gekeler V, Neumann M, Frese G, Handgretinger R, Kardos G, Diddens H and Niethammer D (1990) Susceptibility of multidrug-resistant human leukemia cell lines to human interleukin 2-activated killer cells. *Cancer Res* **50**: 6793–6799

Lautier D, Canitrot Y, Deeley RG and Cole SPC (1996) Multidrug resistance mediated by the multidrug resistance protein (MRP) gene. *Biochem Pharmacol* **52**: 967–977

Ling V and Thompson LH (1974) Reduced permeability in CHO cells as a mechanism of resistance to colchicine. *J Cell Physiol* **83**: 103–116

Liu R and Sharom FJ (1996) Site-directed fluorescence labeling of P-glycoprotein on cysteine residues in the nucleotide binding domains. *Biochemistry* **35**: 11865–11873

Mosman T (1983) Rapid colorimetric assay for cellular growth and survival: application to proliferation and cytotoxicity assays. *J Immunol Methods* **65**: 55–63

Noonan KE, Beck C, Holzmayer TA, Chin JE, Wunder JS, Andrulis IL, Gazdar AF, Willman CL, Griffith B, Von Hoff DD and Roninson IB (1990) Quantitative analysis of MDR1 (multidrug resistance) gene expression in human tumors by polymerase chain reaction. *Proc Natl Acad Sci USA* **87**: 7160–7164

Romsicki, Y and Sharom, FJ (2001) Phospholipid flippase activity of the reconstituted P-glycoprotein multidrug transporter. *Biochemistry* (accepted)

Ruetz S and Gros P (1994) Phosphatidylcholine translocase: a physiological role for the mdr2 gene. *Cell* **77**: 1071–1081

Safa AR, Stern RK, Choi K, Agresti M, Tamai I, Mehta ND and Roninson IB (1990) Molecular basis of preferential resistance to colchicine in multidrug-resistant human cells conferred by Gly-185-Val-185 substitution in P-glycoprotein. *Proc Natl Acad Sci USA* **87**: 7225–7229

Scheffer GL, Winjgaard PLJ, Flens MJ, Izquierdo MA, Slovak ML, Pinedo HM, Meijer CJLM, Clevers HC and Scheper RJ (1995) The drug resistance-related protein LRP is the human major vault protein. *Nature Med* **1**: 578–582

Shapiro AB and Ling V (1997) Positively cooperative sites for drug transport by P-glycoprotein with distinct drug specificities. *Eur J Biochem* **250**: 130–137

Shapiro AB, Fox K, Lam P and Ling V (1999) Stimulation of P-glycoprotein-mediated drug transport by prazosin and progesterone – Evidence for a third drug-binding site. *Eur J Biochem* **259**: 841–850

 *© 2001 Cancer Research Campaign*

Sharom FJ (1997) The P-glycoprotein efflux pump: how does it transport drugs? *J Membr Biol* **160**: 161–175
Sharom FJ, Yu X and Doige CA (1993) Functional reconstitution of drug transport and ATPase activity in proteoliposomes containing partially purified P-glycoprotein. *J Biol Chem* **268**: 24197–24202
Sharom FJ, DiDiodato G, Yu X and Ashbourne KJ (1995) Interaction of the P-glycoprotein multidrug transporter with peptides and ionophores. *J Biol Chem* **270**: 10334–10341
Sharom FJ, Yu X, DiDiodato G and Chu JWK (1996) Synthetic hydrophobic peptides are substrate for P-glycoprotein and stimulate drug transport. *Biochem J* **320**: 421–428
Sharom FJ, Liu R and Romsicki Y (1998a) Spectroscopic and biophysical approaches for studying the structure and function of the P-glycoprotein multidrug transporter. *Biochem Cell Biol* **76**: 695–708
Sharom FJ, Lu P, Liu R and Yu X (1998b) Linear and cyclic peptides as substrates and modulators of P-glycoprotein: peptide binding and effects on drug transport and accumulation. *Biochem J* **333**: 621–630
Sharom FJ, Liu R, Romsicki Y and Lu P (1999) Insights into the structure and substrate interactions of the P-glycoprotein multidrug transporter from spectroscopic studies *Biochim Biophys Acta* **1461**: 327–345
Smit JJ, Schinkel AH, Oude Elferink RP, Groen AK, Wagenaar E, van Deemter L, Mol CA, Ottenhoff R, van der Lugt NM, van Roon MA, van der Valk MA, Offerhaus GJA, Berns AJM and Borst P (1993) Homozygous disruption of the murine mdr2 P-glycoprotein gene leads to a complete absence of phospholipid from bile and to liver disease. *Cell* **75**: 451–462
Smyth MJ, Drasovskis E, Sutton VR and Johnstone RW (1998) The drug efflux protein, P-glycoprotein, additionally protects drug-resistant tumor cells from multiple forms of caspase-dependent apoptosis. *Proc Natl Acad Sci USA* **95**: 7024–7029
Spitaler M, Utz I, Hilbe W, Hofmann J and Grunicke HH (1998) PKC-independent modulation of multidrug resistance in cells with mutant (V185) but not wild-type (G185) P-glycoprotein by bryostatin 1. *Biochem Pharmacol* **56**: 861–869
Thorgeirsson SS, Silverman JA, Gant TW and Marino PA (1991) Multidrug resistance gene family and chemical carcinogenesis. *Pharmacol Ther* **49**: 283–292
Trock B, Leonessa JF and Clarke R (1997) Multidrug resistance in breast cancer: a meta-analysis of MDR1/Pgp170 expression and its possible functional significance. *J Natl Cancer Inst* **89**: 917–931
Vo QD and Gruol DJ (1999) Identification of P-glycoprotein mutations causing a loss of steroid recognition and transport. *J Biol Chem* **274**: 20318–20327

*© 2001 Cancer Research Campaign*
